# Density and refractive index data of binary and ternary mixtures of imidazolium-based ionic liquids, *n*-hexane and organic compounds involved in the kinetic resolution of rac-2-pentanol

**DOI:** 10.1016/j.dib.2018.04.127

**Published:** 2018-05-05

**Authors:** Mercedes G. Montalbán, Mar Collado-González, A. Abel Lozano-Pérez, F. Guillermo Díaz Baños, Gloria Víllora

**Affiliations:** a Department of Chemical Engineering, Faculty of Chemistry, Regional Campus of International Excellence "Campus Mare Nostrum", University of Murcia, 30071 Murcia, Spain; b Department of Physical Chemistry, Faculty of Chemistry, Regional Campus of International Excellence "Campus Mare Nostrum", University of Murcia, 30071 Murcia, Spain; c Department of Biotechnology, Instituto Murciano de Investigación y Desarrollo Agrario y Alimentario (IMIDA), La Alberca (Murcia), 30150, Spain

## Abstract

This data article is related to the subject of the research article “Extraction of Organic Compounds Involved in the Kinetic Resolution of rac-2-Pentanol from n-Hexane by Imidazolium-based Ionic Liquids: Liquid-Liquid Equilibrium” (Montalbán et al., 2018) [1]. It contains experimental data of density and refractive index of binary and ternary mixtures of imidazolium-based ionic liquids, *n*-hexane and organic compounds involved in the kinetic resolution of *rac*-2-pentanol (*rac*-2-pentanol, vinyl butyrate, *rac*-2-pentyl butyrate or butyric acid) measured at 303.15 K and 1 atm. These data are presented as calibration curves which help to determine the composition of the ionic liquid-rich phase knowing its density or refractive index.

**Specifications Table**

**Table:** 

Subject area	*Chemistry*
More specific subject area	*Physical Chemistry*
Type of data	*Figures*
How data was acquired	*RX-5000α refractometer from ATAGO and an Anton Paar DMA-4500 vibrating-tube densimeter*
Data format	*Raw data*
Experimental factors	*Before the measurements, the ionic liquids and the organic compounds were dried under vacuum in the presence of anhydrous phosphorus pentoxide and kept in a desiccator to avoid any moisture absorption*
Experimental features	*The samples were left to equilibrate at 303.15 K for a certain time before an individual measurement*
Data source location	*University of Murcia, Murcia, Spain, Europe*
Data accessibility	*The data are with this article*

**Value of the data**

• *Density and refractive index are two accessible physicochemical properties.*
• *Density and refractive index measurements are fast, accurate and only require small quantities of sample.*
• *Density and refractive index calibration plots of mixtures help to determine the composition of the ionic liquid-rich phase of an unknown sample.*
• *The data can be useful for other researchers investigating the same systems.*

## 1 Data

**Fig. 1:**
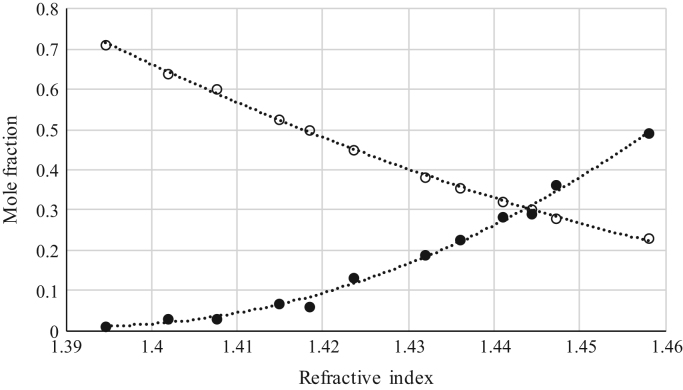
Refractive index calibration curve for ternary mixtures of [bmim^+^][MeSO_4_^−^] + *rac*-2-Pentanol + *n*-hexane at 303.15 K and 0.1 MPa. (○) *n*-hexane (*n*-hexane mole fraction = 40.065 · (refractive index)^2^ – 122.09 · (refractive index) + 93.066; *r*^*2*^ = 0.9987); (•) [bmim^+^][MeSO_4_^−^] ([bmim^+^][MeSO_4_^−^] mole fraction = 114.56 · (refractive index)^2^ -319.21· (refractive index) + 222.36; *r*^*2*^ = 0.9936). Both sets of data (full and empty cycles) correspond to the same experimental run, i.e. refractive index values (*x*-axis) correspond to the refractive index of the ternary mixtures.

**Fig. 2:**
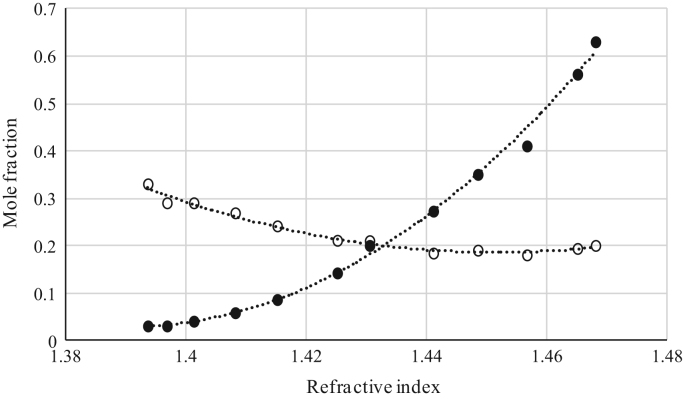
Refractive index calibration curve for ternary mixtures of [bmim^+^][MeSO_4_^−^] + Butyric Acid + *n*-hexane at 303.15 K and 0.1 MPa. (○) *n*-hexane (*n*-hexane mole fraction = 39.974 · (refractive index)^2^ – 116.06 · (refractive index) + 84.423; *r*^*2*^ = 0.9794); (•) [bmim^+^][MeSO_4_^−^] ([bmim^+^][MeSO_4_^−^] mole fraction = 98.065 · (refractive index)^2^ – 272.89 · (refractive index) + 189.87; *r*^*2*^ = 0.9952). Both sets of data (full and empty cycles) correspond to the same experimental run, i.e. refractive index values (*x*-axis) correspond to the refractive index of the ternary mixtures.

**Fig. 3:**
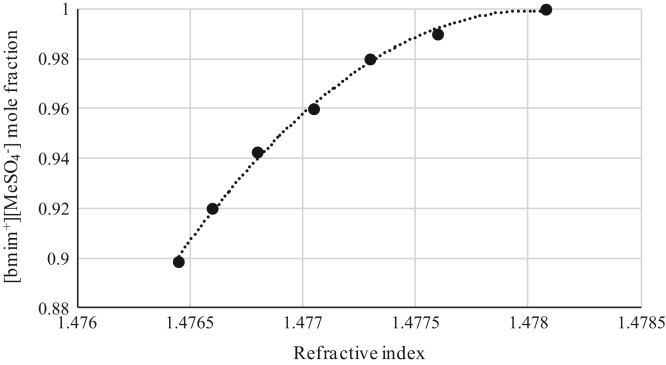
Refractive index calibration curve for binary mixtures of [bmim^+^][MeSO_4_^−^] + *rac*-2-Pentyl Butyrate at 303.15 K and 0.1 MPa. (•) [bmim^+^][MeSO_4_^−^] ([bmim^+^][MeSO_4_^−^] mole fraction = −40205 · (refractive index)^2^ + 118846 · (refractive index) - 87827; *r*^*2*^ = 0.9970). Refractive index values (*x*-axis) correspond to the refractive index of the binary mixtures.

**Fig. 4:**
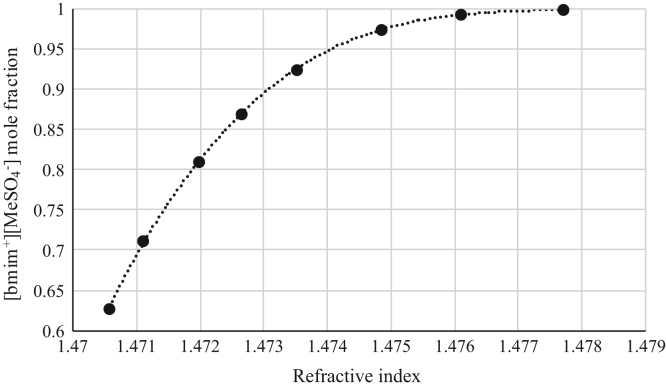
Refractive index calibration curve for binary mixtures of [bmim^+^][MeSO_4_^−^] + Vinyl Butyrate at 303.15 K and 0.1 MPa. (•) [bmim^+^][MeSO_4_^−^] ([bmim^+^][MeSO_4_^−^] mole fraction = 1.1644·10^6^ · (refractive index)^3^ – 5.1603·10^6^ · (refractive index)^2^ + 7.6231·10^6^ · (refractive index) – 3.7537·10^6^; *r*^*2*^ = 0.9998). Refractive index values (*x*-axis) correspond to the refractive index of the binary mixtures.

**Fig. 5:**
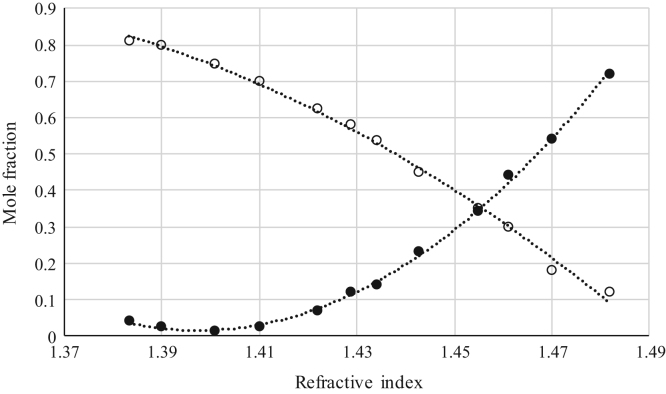
Refractive index calibration curve for ternary mixtures of [emim^+^][Ac^−^] + *rac*-2-Pentanol + *n*-hexane at 303.15 K and 0.1 MPa. (○) *n*-hexane (*n*-hexane mole fraction = − 34.655 · (refractive index)^2^ + 91.879 · (refractive index) - 59.940; *r*^*2*^ = 0.9955); (•) [emim^+^][Ac^−^] ([emim^+^][Ac^−^] mole fraction = 100.13 · (refractive index)^2^ -279.85· (refractive index) + 195.56; *r*^*2*^ = 0.9987). Both sets of data (full and empty cycles) correspond to the same experimental run, i.e. refractive index values (*x*-axis) correspond to the refractive index of the ternary mixtures.

**Fig. 6:**
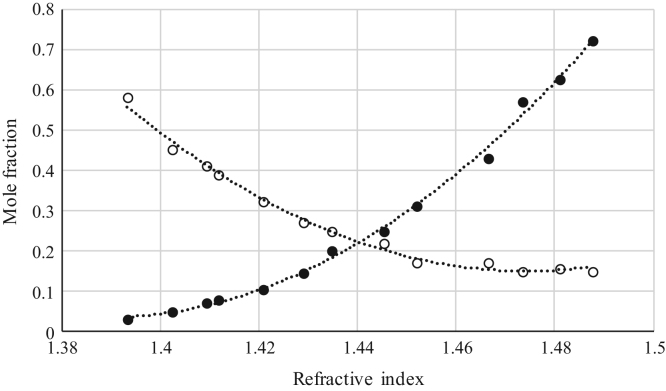
Refractive index calibration curve for ternary mixtures of [emim^+^][Ac^−^] + Butyric Acid + *n*-hexane at 303.15 K and 0.1 MPa. (○) *n*-hexane (*n*-hexane mole fraction = 62.086 · (refractive index)^2^ – 183.06 · (refractive index) + 135.09; *r*^*2*^ = 0.9923); (•) [emim^+^][Ac^−^] ([emim^+^][Ac^−^] mole fraction = 69.912 · (refractive index)^2^ – 194.18 · (refractive index) + 134.86; *r*^*2*^ = 0.9964). Both sets of data (full and empty cycles) correspond to the same experimental run, i.e. refractive index values (*x*-axis) correspond to the refractive index of the ternary mixtures.

**Fig. 7:**
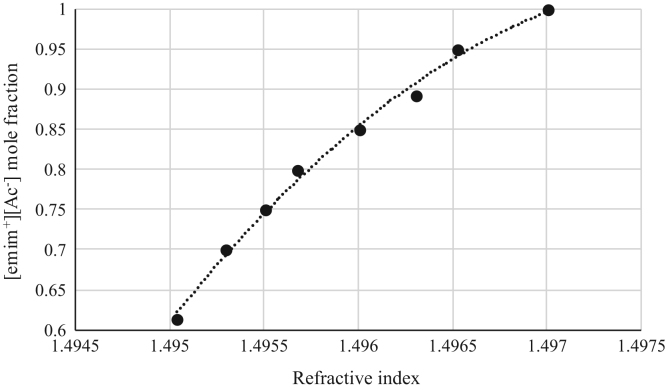
Refractive index calibration curve for binary mixtures of [emim^+^][Ac^−^] + *rac*-2-Pentyl Butyrate at 303.15 K and 0.1 MPa. (•) [emim^+^][Ac^−^] ([emim^+^][Ac^−^] mole fraction = −50730 · (refractive index)^2^ + 151977 · (refractive index) + 113822; *r*^*2*^ = 0.9945). Refractive index values (*x*-axis) correspond to the refractive index of the binary mixtures.

**Fig. 8:**
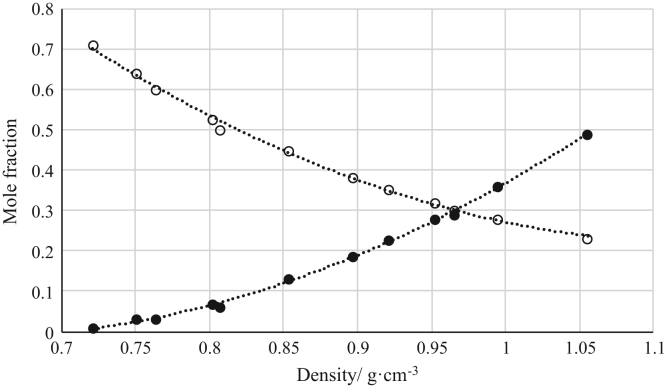
Density calibration curve for ternary mixtures of [bmim^+^][MeSO_4_^−^] + *rac*-2-Pentanol + *n*-hexane at 303.15 K and 0.1 MPa. (○) *n*-hexane (*n*-hexane mole fraction = 2.7657 · (density)^2^ – 6.2963 · (density) + 3.8019; *r*^*2*^ = 0.9966); (•) [bmim^+^][MeSO_4_^−^] ([bmim^+^][MeSO_4_^−^] mole fraction = 2.7572 · (density)^2^ – 3.4520 · (density) + 1.0626; *r*^*2*^ = 0.9984). Both sets of data (full and empty cycles) correspond to the same experimental run, i.e. density values (*x*-axis) correspond to the density of the ternary mixtures.

**Fig. 9:**
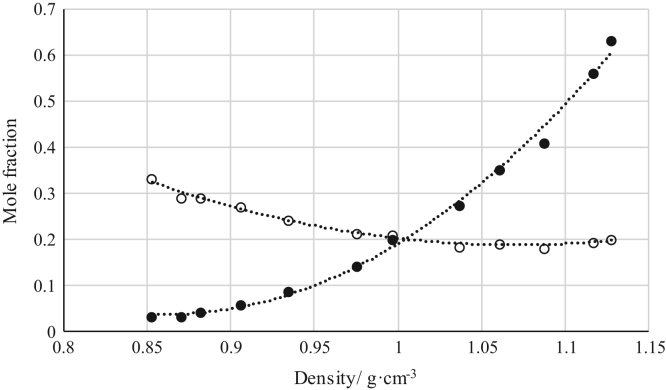
Density calibration curve for ternary mixtures of [bmim^+^][MeSO_4_^−^] + Butyric Acid + *n*-hexane at 303.15 K and 0.1 MPa. (○) *n*-hexane (*n*-hexane mole fraction = 2.9034 · (density)^2^ – 6.2131 · (density) + 3.5106; *r*^*2*^ = 0.9857); (•) [bmim^+^][MeSO_4_^−^] ([bmim^+^][MeSO_4_^−^] mole fraction = 8.0635 · (density)^2^ – 13.900 · (density) + 6.0259; *r*^*2*^ = 0.9950). Both sets of data (full and empty cycles) correspond to the same experimental run, i.e. density values (*x*-axis) correspond to the density of the ternary mixtures.

**Fig. 10:**
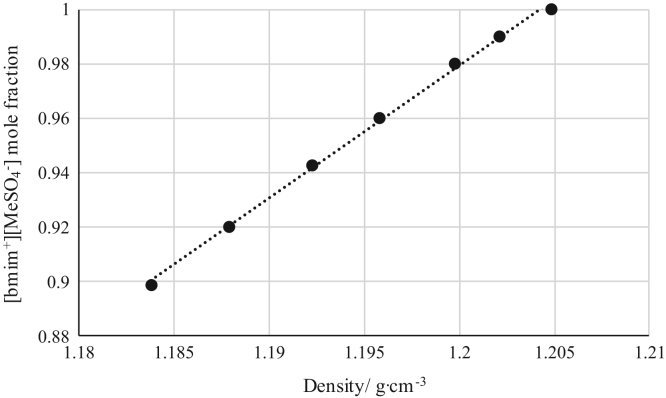
Density calibration curve for binary mixtures of [bmim^+^][MeSO_4_^−^] + *rac*-2-Pentyl Butyrate at 303.15 K and 0.1 MPa. (•) [bmim^+^][MeSO_4_^−^] ([bmim^+^][MeSO_4_^−^] mole fraction = 4.8796 · (density) – 4.8760; *r*^*2*^ = 0.9979). Density values (*x*-axis) correspond to the density of the binary mixtures.

**Fig. 11:**
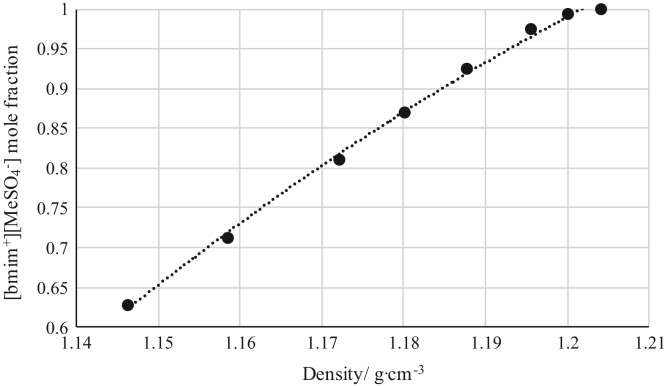
Density calibration curve for binary mixtures of [bmim^+^][MeSO_4_^−^] + Vinyl Butyrate at 303.15 K and 0.1 MPa. (•) [bmim^+^][MeSO_4_^−^] ([bmim^+^][MeSO_4_^−^] mole fraction = – 25.013 · (density)^2^ + 65.532 · (density) – 41.628; *r*^*2*^ = 0.9969). Density values (*x*-axis) correspond to the density of the binary mixtures.

**Fig. 12:**
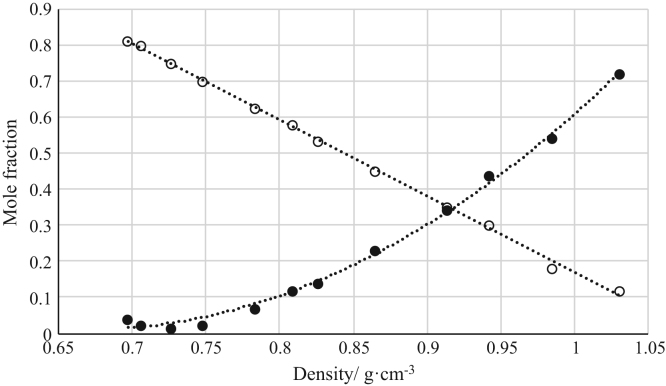
Density calibration curve for ternary mixtures of [emim^+^][Ac^−^] + *rac*-2-Pentanol + *n*-hexane at 303.15 K and 0.1 MPa. (○) *n*-hexane (*n*-hexane mole fraction = − 2.1128 · (density) + 2.2820; *r*^*2*^ = 0.9984); (•) [emim^+^][Ac^−^] ([emim^+^][Ac^−^] mole fraction = 5.4668 · (density)^2^ – 7.3187 · (density) + 2.4604; *r*^*2*^ = 0.9961). Both sets of data (full and empty cycles) correspond to the same experimental run, i.e. density values (*x*-axis) correspond to the density of the ternary mixtures.

**Fig. 13:**
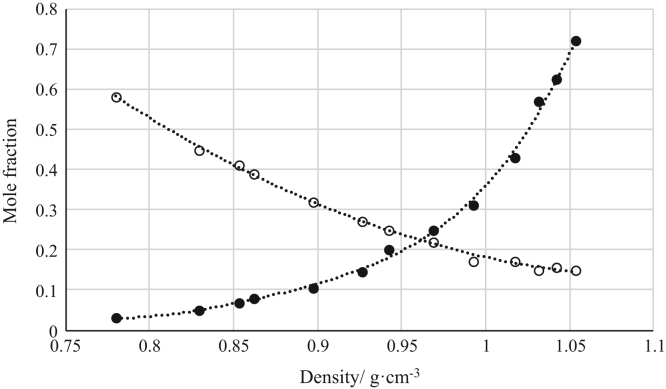
Density calibration curve for ternary mixtures of [emim^+^][Ac^−^] + Butyric Acid + *n*-hexane at 303.15 K and 0.1 MPa. (○) *n*-hexane (*n*-hexane mole fraction = 4.0896 · (density)^2^ – 9.0935 · (density) + 5.1860; *r*^*2*^ = 0.9976); (•) [emim^+^][Ac^−^] ([emim^+^][Ac^−^] mole fraction = 244.53 · (density)^4^ - 837.93· (density)^3^ + 1079.6 · (density)^2^ – 618.96 · (density) + 133.12; *r*^*2*^ = 0.9969). Both sets of data (full and empty cycles) correspond to the same experimental run, i.e. density values (*x*-axis) correspond to the density of the ternary mixtures.

**Fig. 14:**
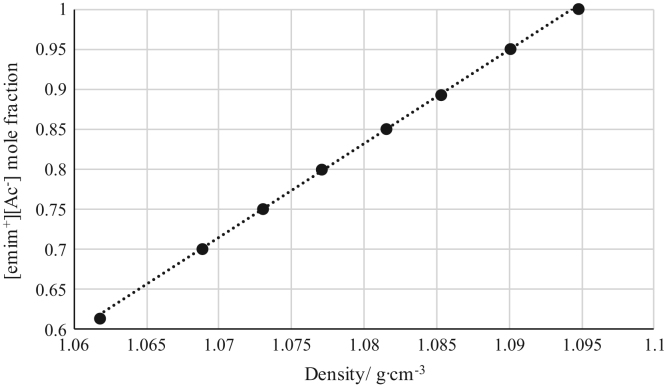
Density calibration curve for binary mixtures of [emim^+^][Ac^−^] + *rac*-2-Pentyl Butyrate at 303.15 K and 0.1 MPa. (•) [emim^+^][Ac^−^] ([emim^+^][Ac^−^] mole fraction = 11.731 · (density) – 11.838; *r*^*2*^ = 0.9996). Density values (*x*-axis) correspond to the density of the binary mixtures.

The data reported include refractive index (Fig. 1, Fig. 2, Fig. 3, Fig. 4, Fig. 5, Fig. 6, Fig. 7) and density (Fig. 8, Fig. 9, Fig. 10, Fig. 11, Fig. 12, Fig. 13, Fig. 14) calibration curves of binary and ternary mixtures involving ionic liquids, *n*-hexane and an organic compound present in the racemic resolution of *rac*-2-pentanol. Ternary systems correspond to Fig. 1, Fig. 2, Fig. 5, Fig. 6, Fig. 8, Fig. 9, Fig. 12, Fig. 13. When the ternary mixture was not possible because the insolubility of the components, binary systems were analyzed (Fig. 3, Fig. 4, Fig. 7, Fig. 10, Fig. 11, Fig. 14).

Table 1, Table 2, Table 3, Table 4, Table 5, Table 6, Table 7 collect the composition (in terms of mole fraction) of the binary and ternary mixtures used to obtain the calibration plots shown in Fig. 1, Fig. 2, Fig. 3, Fig. 4, Fig. 5, Fig. 6, Fig. 7, Fig. 8, Fig. 9, Fig. 10, Fig. 11, Fig. 12, Fig. 13, Fig. 14 and experimental refractive index and density data.

**Table 1:** Composition (in mole fraction), refractive index and density (g cm^−3^) of ternary mixtures [bmim^+^][MeSO_4_^−^] (1) + *rac*-2-Pentanol (2) + *n*-hexane (3) at 303.15 K and 0.1 MPa.^a^

***x***_***1***_	***x***_***2***_	***x***_***3***_	**Refractive index**	**Density/ g cm**^**−3**^
0.0101	0.2799	0.7100	1.39467	0.72133
0.0301	0.3301	0.6398	1.40203	0.75044
0.0300	0.3701	0.5999	1.40758	0.76340
0.0673	0.4064	0.5263	1.41502	0.80253
0.0599	0.4400	0.5001	1.41849	0.80670
0.1302	0.4199	0.4499	1.42373	0.85322
0.1884	0.4291	0.3825	1.43201	0.89715
0.2269	0.4198	0.3533	1.43590	0.92136
0.2805	0.4002	0.3193	1.44099	0.95206
0.2899	0.4101	0.3000	1.44439	0.96596
0.3600	0.3599	0.2801	1.44729	0.99515
0.4903	0.2793	0.2304	1.45803	1.05542

a Standard uncertainties *u* are *u(ρ)*=0.00005 g/cm^3^ with *u*(*T*^*ρ*^) = 0.03 K and *u(n)* = 0.00004 (nD) with *u*(*T*^*n*^) = 0.02 K. Standard uncertainty in pressure was *u*(*P*)=10 kPa.

**Table 2:** Composition (in mole fraction), refractive index and density (g cm^−3^) of ternary mixtures [bmim^+^][MeSO_4_^−^] (1) + Butyric Acid (2) + *n*-hexane (3) at 303.15 K and 0.1 MPa.^a^

***x***_***1***_	***x***_***2***_	***x***_***3***_	**Refractive index**	**Density/ g cm**^**−3**^
0.0301	0.6399	0.3300	1.39345	0.85307
0.0298	0.6801	0.2901	1.39664	0.87028
0.0399	0.6702	0.2899	1.40110	0.88173
0.0579	0.6733	0.2688	1.40801	0.90594
0.0857	0.6731	0.2412	1.41498	0.93437
0.1419	0.6473	0.2108	1.42499	0.97500
0.2003	0.5900	0.2097	1.43038	0.99689
0.2724	0.5438	0.1838	1.44102	1.03678
0.3505	0.4594	0.1901	1.44841	1.06096
0.4097	0.4103	0.1800	1.45658	1.08724
0.5615	0.2449	0.1936	1.46503	1.11683
0.6300	0.1702	0.1998	1.46801	1.12762

a Standard uncertainties *u* are *u(ρ)* = 0.00005 g/cm^3^ with *u*(*T*^*ρ*^) = 0.03 K and *u(n)*=0.00004 (nD) with *u*(*T*^*n*^) = 0.02 K. Standard uncertainty in pressure was *u*(*P*) = 10 kPa.

**Table 3:** Composition (in mole fraction), refractive index and density (g cm^−3^) of binary mixtures [bmim^+^][MeSO_4_^−^] (1) + *rac*-2-Pentyl Butyrate (2) at 303.15 K and 0.1 MPa.^a^

***x***_***1***_	***x***_***2***_	**Refractive index**	**Density/ g cm**^**−3**^
0.8986	0.1014	1.47645	1.18376
0.9199	0.0801	1.47660	1.18785
0.9426	0.0574	1.47679	1.19222
0.9602	0.0398	1.47705	1.19576
0.9800	0.0200	1.47731	1.19973
0.9901	0.0099	1.47760	1.20208
1.0000	0.0000	1.47808	1.20481

a Standard uncertainties *u* are *u(ρ)*=0.00005 g/cm^3^ with *u*(*T*^*ρ*^) = 0.03 K and *u(n)* = 0.00004 (nD) with *u*(*T*^*n*^) = 0.02 K. Standard uncertainty in pressure was *u*(*P*)=10 kPa.

**Table 4:** Composition (in mole fraction), refractive index and density (g cm^−3^) of binary mixtures [bmim^+^][MeSO_4_^−^] (1) + Vinyl Butyrate (2) at 303.15 K and 0.1 MPa.^a^

***x***_***1***_	***x***_***2***_	**Refractive index**	**Density/ g cm**^**−3**^
0.6275	0.3725	1.47057	1.14619
0.7120	0.2880	1.47110	1.15841
0.8105	0.1895	1.47198	1.17203
0.8700	0.1300	1.47265	1.18001
0.9249	0.0751	1.47352	1.18761
0.9750	0.0250	1.47485	1.19544
0.9941	0.0059	1.47610	1.19996
1.0000	0.0000	1.47771	1.20401

a Standard uncertainties *u* are *u(ρ)*=0.00005 g/cm^3^ with *u*(*T*^*ρ*^) = 0.03 K and *u(n)* = 0.00004 (nD) with *u*(*T*^*n*^) = 0.02 K. Standard uncertainty in pressure was *u*(*P*) = 10 kPa.

**Table 5:** Composition (in mole fraction), refractive index and density (g cm^−3^) of ternary mixtures [emim^+^][Ac^−^] (1) + *rac*-2-Pentanol (2) + *n*-hexane (3) at 303.15 K and 0.1 MPa.^a^

***x***_***1***_	***x***_***2***_	***x***_***3***_	**Refractive index**	**Density/ g cm**^**−3**^
0.0401	0.1499	0.8100	1.38334	0.69709
0.0235	0.1854	0.7911	1.39001	0.70591
0.0138	0.2387	0.7475	1.40102	0.72602
0.0251	0.2763	0.6986	1.41001	0.74743
0.0672	0.3099	0.6229	1.42200	0.78293
0.1200	0.2999	0.5801	1.42885	0.80866
0.1401	0.3249	0.5350	1.43403	0.82565
0.2299	0.3201	0.4500	1.44268	0.86497
0.3419	0.3060	0.3521	1.45501	0.91348
0.4402	0.2597	0.3001	1.46115	0.94176
0.5400	0.2801	0.1799	1.46990	0.98449
0.7201	0.1600	0.1199	1.48186	1.03085

a Standard uncertainties *u* are *u(ρ)* = 0.00005 g/cm^3^ with *u*(*T*^*ρ*^) = 0.03 K and *u(n)* = 0.00004 (nD) with *u*(*T*^*n*^) = 0.02 K. Standard uncertainty in pressure was *u*(*P*) = 10 kPa.

**Table 6:** Composition (in mole fraction), refractive index and density (g cm^−3^) of ternary mixtures [emim^+^][Ac^−^] (1) + Butyric Acid (2) + *n*-hexane (3) at 303.15 K and 0.1 MPa.^a^

***x***_***1***_	***x***_***2***_	***x***_***3***_	**Refractive index**	**Density/ g cm**^**−3**^
0.0301	0.3900	0.5799	1.39324	0.77996
0.0498	0.4999	0.4503	1.40255	0.82936
0.0702	0.5301	0.4097	1.40931	0.85353
0.0800	0.5302	0.3898	1.41179	0.86243
0.1044	0.5746	0.3210	1.42101	0.89765
0.1457	0.5846	0.2697	1.42897	0.92644
0.2003	0.5497	0.2500	1.43477	0.94224
0.2499	0.5300	0.2201	1.44525	0.96906
0.3125	0.5149	0.1726	1.45200	0.99249
0.4297	0.4001	0.1702	1.46647	1.01731
0.5702	0.2802	0.1496	1.47341	1.03134
0.6243	0.2196	0.1561	1.48099	1.04242
0.7200	0.1297	0.1503	1.48763	1.05407

a Standard uncertainties *u* are *u(ρ)* = 0.00005 g/cm^3^ with *u*(*T*^*ρ*^) = 0.03 K and *u(n)* = 0.00004 (nD) with *u*(*T*^*n*^) = 0.02 K. Standard uncertainty in pressure was *u*(*P*) = 10 kPa.

**Table 7:** Composition (in mole fraction), refractive index and density (g cm^−3^) of binary mixtures [emim^+^][Ac^−^] (1) + *rac*-2-Pentyl Butyrate (2) at 303.15 K and 0.1 MPa.^a^

*x*_*1*_	*x*_*2*_	Refractive index	Density/ g cm^−3^
0.6131	0.3869	1.49504	1.06175
0.7001	0.2999	1.49530	1.06881
0.7502	0.2498	1.49551	1.07298
0.7993	0.2007	1.49568	1.07704
0.8498	0.1502	1.49601	1.08149
0.8924	0.1076	1.49631	1.08527
0.9501	0.0499	1.49653	1.09005
1.0000	0.0000	1.49701	1.09474

a Standard uncertainties *u* are *u(ρ)* = 0.00005 g/cm^3^ with *u*(*T*^*ρ*^) = 0.03 K and *u(n)* = 0.00004 (nD) with *u*(*T*^*n*^) = 0.02 K. Standard uncertainty in pressure was *u*(*P*)=10 kPa.

## 2 Experimental design, materials and methods

### 2.1 Materials

The ionic liquids 1-butyl-3-methylimidazolium methylsulphate, [bmim^+^][MeSO_4_^−^] (purity > 0.99), and 1-ethyl-3-methylimidazolium acetate, [emim^+^][Ac^−^] (purity > 0.95) were provided from Iolitec. All other chemicals were supplied by Sigma-Aldrich (purity > 0.98). The structures and molecular weight are depicted in [1]. The same samples of butyric acid, vinyl butyrate, *rac*-2-pentyl butyrate, and *rac*-2-pentanol with the same physical properties as in [2] were used.

### 2.2 Sample preparation

The ionic liquids and the organic compounds were dried under vacuum in the presence of anhydrous phosphorus pentoxide and kept in a desiccator to avoid any moisture absorption. The water contents of the ionic liquids and the organic compounds was determined with a Karl Fischer coulometric titrator (Metrohm, 831 KF). The values of the water content were low in all cases (*w* < 0.001). The standard uncertainty, *u*, of the water content measurements was *u*(*w*) = 0.1 µg/mL. The ternary and binary mixtures studied were prepared in glass vials by weighing each component on a Sartorius BF121S balance with a precision of 10^−4^ g, in order to obtain a mixture with a known composition. The standard uncertainty, *u*, of the mole fraction is *u*(*x*) = 0.0003.

### 2.3 Refractive index measurement

The index of refraction of the ionic liquid-rich phase was measured using a RX-5000α refractometer from ATAGO (*λ* = 589 nm). Earlier, the refractive index of the ternary mixtures was correlated with their respective concentrations by calibration curves in order to obtain the component concentrations in this phase. Standard uncertainties were *u*(*T*^*n*^) = 0.02 K and *u*(*n*) = 0.00004 (nD).

### 2.4 Density measurement

The density of the ionic liquid-rich phase, which was also correlated with concentration, was measured with an Anton Paar DMA-4500 vibrating-tube densimeter. In this case, standard uncertainties were *u*(*T*^*ρ*^) = 0.03 K and *u*(*ρ*) = 0.00005 g/cm^3^.

Therefore, the calibration method was used to measure the refractive index and density of the ionic liquid-rich phase at 303.15 K by preparing calibration plots (Fig. 1, Fig. 2, Fig. 3, Fig. 4, Fig. 5, Fig. 6, Fig. 7, Fig. 8, Fig. 9, Fig. 10, Fig. 11, Fig. 12, Fig. 13, Fig. 14) of the refractive index and density from several known phase compositions. The standard uncertainty, *u*, of the concentration (in terms of mole fraction) predicted by the empirical correlations of refractive index and density is *u*(*x*) = 0.003.

After use, the ionic liquids have been regenerated following procedures previously published in the literature with other organic solvents [3]. Briefly, organic compound (2-pentanol, vinyl butyrate, 2-pentyl butyrate or butyric acid)/hexane mixtures can be removed from the ionic liquids on a rotary evaporator at 80 °C and 80 mbar. The ionic liquids were kept in a desiccator until reuse.
