# Supplementary material for: Density and refractive index data of binary and ternary mixtures of imidazolium-based ionic liquids, n-hexane and organic compounds involved in the kinetic resolution of rac-2-pentanol
Source: Data Brief. 2018 May 5;19:134–44. doi: 10.1016/j.dib.2018.04.127 (PMC5992958; doi:10.1016/j.dib.2018.04.127)
Supplement: Supplementary file 1 — Supplementary material [file mmc1.docx]

Authors declare no conflict of interest.

Dr. Mercedes G. Montalbán
